# Nanog-driven cell-reprogramming and self-renewal maintenance in *Ptch1*^+/−^ granule cell precursors after radiation injury

**DOI:** 10.1038/s41598-017-14506-6

**Published:** 2017-10-27

**Authors:** Barbara Tanno, Simona Leonardi, Gabriele Babini, Paola Giardullo, Ilaria De Stefano, Emanuela Pasquali, Anna Saran, Mariateresa Mancuso

**Affiliations:** 1 Laboratory of Biomedical Technologies, Italian National Agency for New Technologies, Energy and Sustainable Economic Development (ENEA), Rome, Italy; 2 Department of Physics, University of Pavia, Pavia, Italy; 3 Department of Radiation Physics, Guglielmo Marconi University, Rome, Italy; 4 Department of Sciences, Roma Tre University, Rome, Italy

## Abstract

Medulloblastoma (MB) is the most common pediatric brain tumor, comprising four distinct molecular variants, one of which characterized by activation of the Sonic Hedgehog (SHH) pathway, driving 25–30% of sporadic MB. SHH-dependent MBs arise from granule cell precursors (GCPs), are fatal in 40–70% of cases and radioresistance strongly contributes to poor prognosis and tumor recurrence. *Patched1* heterozygous (*Ptch1* ^+/−^) mice, carrying a germ-line heterozygous inactivating mutation in the *Ptch1* gene, the Shh receptor and negative regulator of the pathway, are uniquely susceptible to MB development after radiation damage in neonatal cerebellum. Here, we irradiated *ex-vivo* GCPs isolated from cerebella of neonatal WT and *Ptch1* ^+/−^ mice. Our results highlight a less differentiated status of *Ptch1-*mutated cells after irradiation, influencing DNA damage response. Increased expression levels of pluripotency genes Nanog, Oct4 and Sal4, together with greater clonogenic potential, clearly suggest that radiation induces expansion of the stem-like cell compartment through cell-reprogramming and self-renewal maintenance, and that this mechanism is strongly dependent on Nanog. These results contribute to clarify the molecular mechanisms that control radiation-induced Shh-mediated tumorigenesis and may suggest Nanog as a potential target to inhibit for adjuvant radiotherapy in treatment of SHH-dependent MB.

## Introduction

Medulloblastoma (MB) is the most common childhood malignancy of the central nervous system (CNS) and, despite aggressive multimodal therapy with surgery, radiation, and chemotherapy, its 5-year survival rates have only approached >60%^1^. MBs arise from granule cell precursors (GCPs), a transient population of progenitors that, in normal conditions, give rise to granule neurons of the cerebellum^2^.

In 2003, for the first time, a CD133^+^ cell subpopulation with stem cell properties was isolated *in vitro* from human brain tumors. The CD133^+^ cancer stem cell (CSC) fraction, ranging from 19 to 29% in highly aggressive glioblastomas and from 6 to 21% in MBs, correlated closely with an *in vitro* primary sphere formation assay^3^. There are many genetic pathways and elements involved in the control of self-renewal and differentiation of normal SCs as well as CSCs, e.g. PI3K/Akt, JAK/STAT, Wnt/β-catenin, Sonic Hedgehog (SHH), Notch, NF-κB and ABC superfamily among others^4^. In particular, SHH signaling sustains embryonic and postnatal development of neural SCs of the forebrain subventricular zone and hippocampus^5–9^. Moreover, human gliomas display a stemness signature, and SHH-GLI signaling regulates the expression of stemness genes (e.g. Nanog, Oct4, Sox2, CD133) and the self-renewal of CD133(+) glioma cancer stem cells^10,11^. Another SHH target organ is the cerebellum, where SHH is critically required to keep transit-amplifying GCPs in an undifferentiated state and promote their proliferation^12–14^. However, constitutive activation of this pathway can cause MB^15^.

Mice with one copy of the *Ptch1* gene knocked out (*Ptch1* ^+/−^), characterized by aberrant activation of the Shh signaling pathway, are developmentally nearly normal but show a marked predisposition to development of tumors, including MB. Remarkably, they are very radiosensitive^16,17^. Our previous work has shown that irradiation of *Ptch1* ^+/−^ mice during postnatal days 1–10, when GCPs are highly proliferative, significantly increases MB frequency^18,19^.

Self-renewal and pluripotency are two fundamental characteristics of SCs, and are controlled by diverse regulatory factors, including microRNAs (miRNAs). miR-125 isoforms promote neural conversion of human embryonic stem cells. Boissart *et al*.^20^ have shown that antagonizing miR-125 isoforms, independently or in combination, compromised to the same extent the efficiency of neural induction and that both are able to target Lin28, an RNA-binding protein promoting the proliferative capacity of neural progenitor cells in brain development^21^. More recently, miR-125b has been shown to target Lin28 during mouse embryoid body formation^22^. In addition, when both miR-125 isoforms were antagonized simultaneously, expression of all pluripotency markers (including OCT4 and NANOG) was significantly induced^20^.

In our laboratory, we recently identified a subset of miRNAs (i.e. let-7 family and mir-17∼92 cluster), controlling different biological functions, whose expression was altered in GCPs by radiation alone or in combination with Shh-deregulation^23^. An increased level of miR-17 and an associated decreased expression of Let-7 family members after irradiation, suggested an interaction of radiation with Shh-deregulation in sustaining a phenotype shift toward stemness. Importantly, their deregulation persisted in radio-induced MB^23^.

In the current work, through extensive characterization of GCPs response to radiation, we show for the first time that irradiated *Ptch1* ^+/−^ GCPs re-acquire the capacity for long-term self-renewal, reprogramming to a less differentiated cell type through Nanog activation and, importantly, that this process may drive radiogenic Shh-mediated MB tumorigenesis.

## Results

### DNA damage response (DDR) in WT and *Ptch1*^+/−^ GCPs

**Figure 1:**
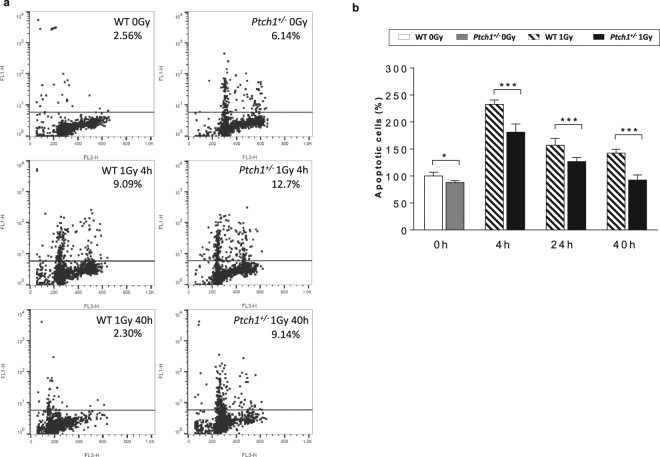
(**a**) Temporal determination of γ-H2AX positive cells by Flow Cytometric Analysis in unirradiated and irradiated WT and *Ptch1* ^+/−^ GCPs. Representative results from three independent experiments are shown. (**b**) Apoptotic rate measured by Caspase-Glo® 3/7 Assay in unirradiated GCPs of both genotypes at different times after irradiation. The results of triplicate assays are expressed as mean ± SD relative to WT GCPs, taken as 100. ***P < 0.001.

GCPs isolated from cerebella of WT and *Ptch1* ^+/−^ mice at P2 and irradiated *ex-vivo* with 1-Gy of X-rays or sham-irradiated, were analyzed by flow cytometry at 4, 24 and 40 hours after irradiation to check for accumulation of γ-H2AX, a typical biomarker for DNA double-strand breaks (DSBs). As shown in Fig. 1a, sham-irradiated *Ptch1* ^+/−^ GCPs showed a higher level of γ-H2AX-positive cells compared with WT GCPs, confirming that Shh signaling pathway deregulation *per se* promotes genetic instability^24^. Consistent with the well-recognized role of ionizing radiation as inductor of DNA DSBs^25^, the accumulation of γ-H2AX strongly increased after irradiation, irrespective of GCPs *Ptch1* genotype. To understand how DNA DBSs induction was paralleled by apoptosis in the two cell populations, we quantified apoptosis of cells by caspase-3/7 assay. Despite generally higher levels of DNA DSBs, we found that *Ptch1* ^+/−^ GCPs were less prone to radiation-induced apoptosis at all time points analyzed (Fig. 1b). Forty hours after irradiation, *Ptch1* ^+/−^ GCPs showed a percentage of apoptotic cells comparable to unirradiated *Ptch1* ^+/−^ cells. Notably, at this experimental time, *Ptch1* ^+/−^ GCPs maintain a high level of γ-H2AX-positive cells triggering persistence of genomic instability. In contrast, WT GCPs at 40 h have a persistent higher percentage of apoptotic cells compared with unirradiated counterparts (*P* < 0.0001), notwithstanding similar DSBs levels.

**Figure 2:**
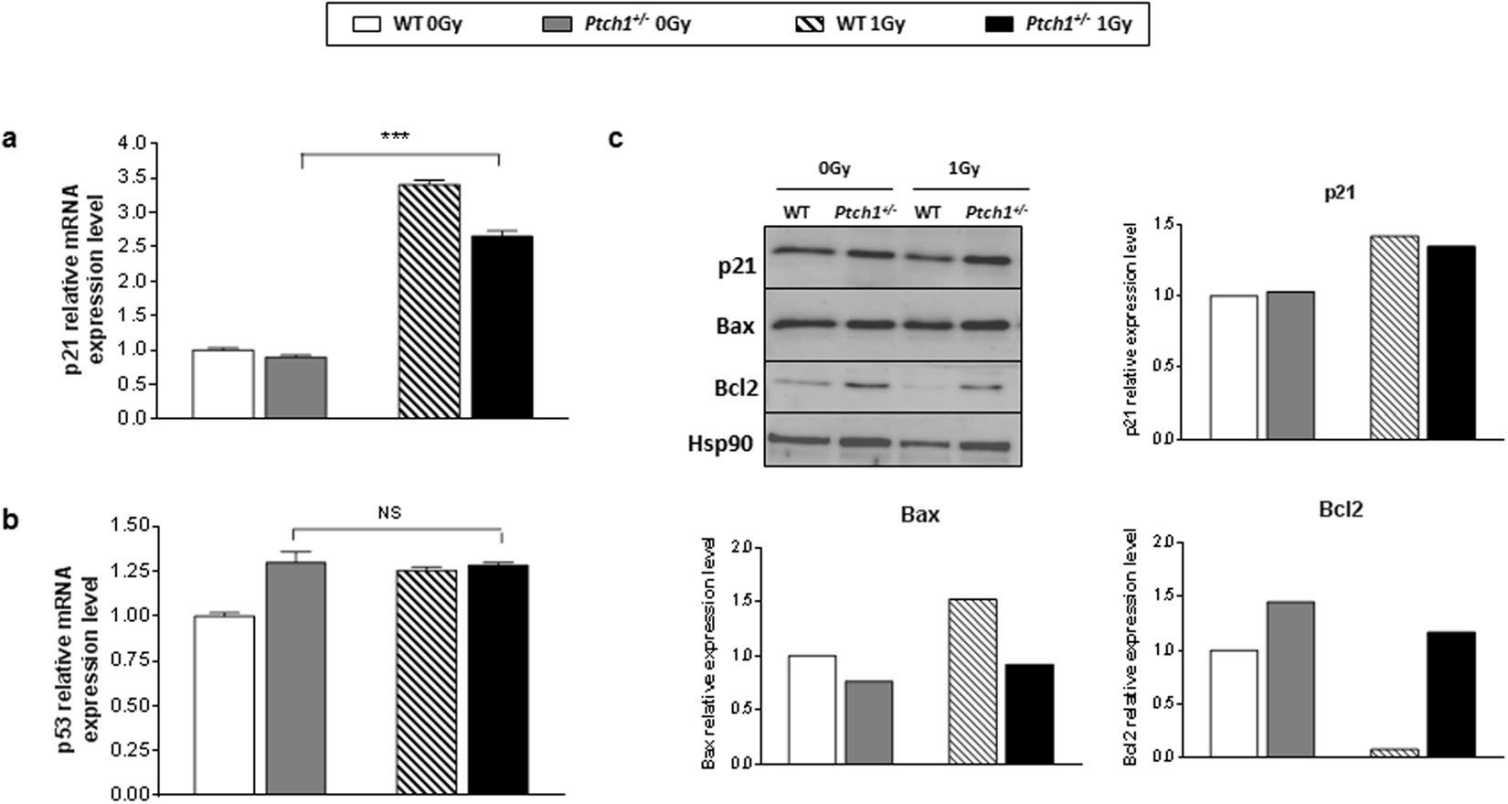
Relative mRNA expression levels of p21 (**a**) and p53 (**b**) in unirradiated WT and *Ptch1* ^+/−^ GCPs and in irradiated GCPs 4 hours after irradiation with 1 Gy of X-rays. (**c**) Western blot analysis and relative densitometry of p21, Bax and Bcl2 expression. Band intensities were normalized against Hsp90. ***P < 0.001. Uncropped Western blot gels related to this figure are displayed in Suppl. Fig. S1.

The DDR can differ depending on the differentiation status of cells, and this difference is ascribable to p21 activation and, at least in part, to its ability to inhibit p53 then impending apoptosis^26^. To verify this mechanism in our experimental system, we checked the expression of p21 and p53 in sham-irradiated and irradiated WT and *Ptch1* ^+/−^ GCPs. The expression level of p21 mRNA (Fig. 2a) and protein (Fig. 2c) significantly increased after irradiation in both cell lineages, but this was not associated to transcriptional activation of p53 in *Ptch1* ^+/−^ GCPs (Fig. 2b). Conversely, in irradiated WT GCPs we found a statistically significant increase of p53 mRNA with respect to sham-irradiated WT cells (Fig. 2b; *P* < 0.05). Noteworthy, the higher expression level of the survival factor Bcl2 and the concomitant lower expression of Bax, a pro-apoptotic Bcl-2-family protein, in irradiated *Ptch1* ^+/−^ GCPs compared with WT cells (Fig. 2c), is in line with the lower degree of apoptosis found in *Ptch1-*mutant cells. This result is supported by data obtained through propidium iodide staining followed by flow cytometric analysis (Supplementary Information file (Table S1)), showing a lower percentage of cell death (hypodiploid peak) and a higher proliferation rate of *Ptch1* ^+/−^ GCPs compared with normal cells, in all experimental conditions.

Altogether, these results underline a different DDR of *Ptch1* ^+/−^ compared with WT GCPs, suggesting a less differentiated status of *Ptch1-*mutant cells and, importantly, that radiation exposure potentiates this difference.

### Cell-reprogramming and cell renewal of *Ptch1*^+/−^ GCPs is induced by irradiation

**Figure 3:**
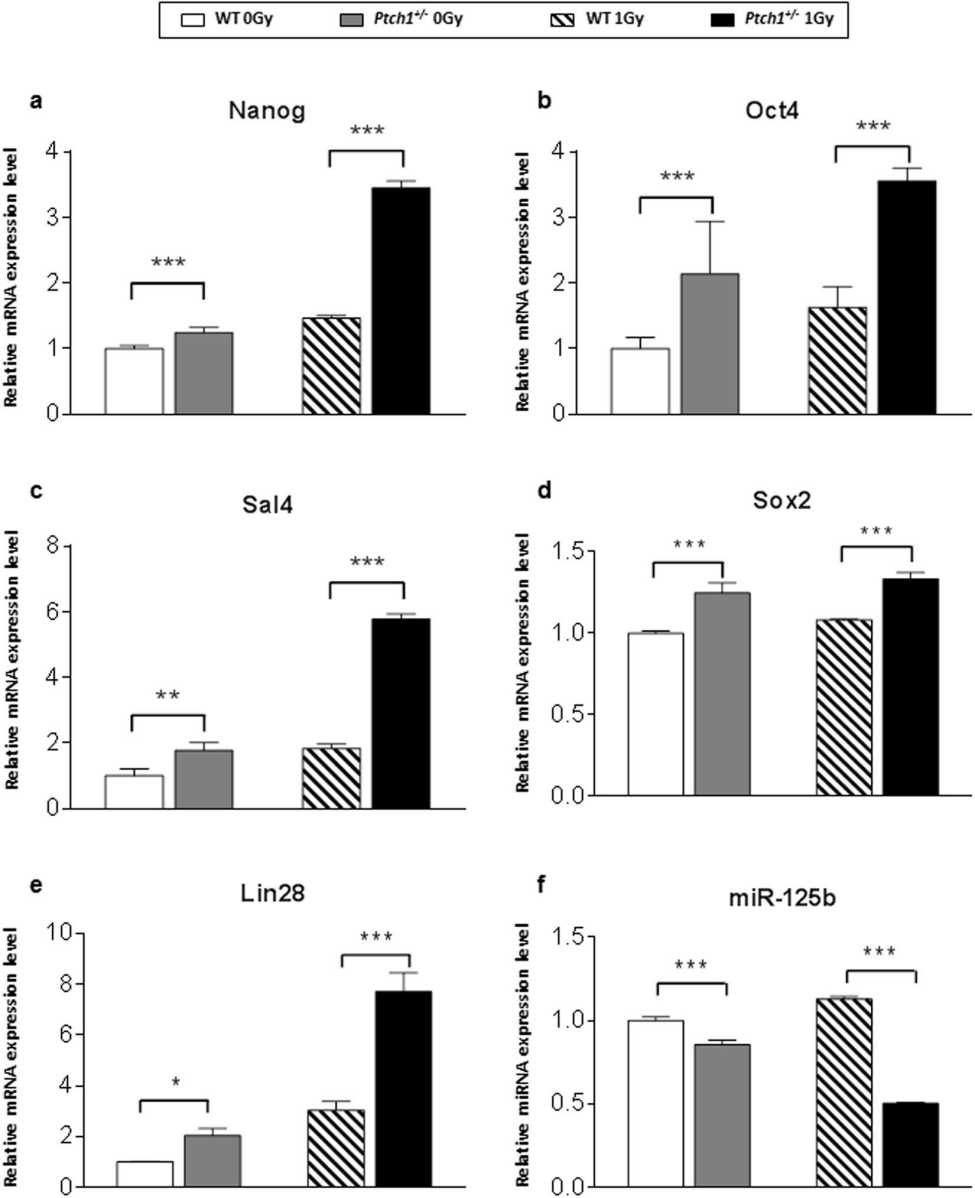
Relative mRNA expression levels of stem cell transcription factors Nanog (**a**), Oct4 (**b**), Sal4 (**c**) and Sox2 (**d**) in unirradiated WT and *Ptch1* ^+/−^ GCPs and in irradiated GCPs 4 hours after irradiation with 1 Gy of X-rays. (**e**) Relative mRNA expression levels of Lin28, a RNA binding protein, and of the neurogenic miR-125b (**f**). Results are expressed as mean ± SD of three biological replicates. Expression levels of WT GCPs are taken as 1. *P ≤ 0.05; **P ≤ 0.01; *** *P* ≤ 0.001.

To explore potential differences in cell differentiation state due to *Ptch1* allelic status, we first examined the expression of transcription factors that are known to induce stem-like properties in differentiated cells, i.e., Nanog, Oct4, Sal4 and Sox2^27^. Unirradiated *Ptch1* ^+/−^ GCPs showed a general statistically significant increase in the expression of all genes analyzed compared to WT GCPs, confirming that increased Shh signalling *per se* causes expansion of the stem cell pool (Fig. 3a–d)^28^. Although irradiation significantly increased expression levels of Nanog, Oct4 and Sal4 in WT GCPs (Fig. 3a–c), their expression was dramatically higher in irradiated *Ptch1* ^+/−^ compared with WT GCPs at 4 hours post-irradiation (Fig. 3a–c), implying that Shh deregulation and irradiation synergize in driving expansion of a highly undifferentiated cell population. Interestingly, no modulation of Sox2 expression level was detected after irradiation, irrespective of genotype (Fig. 3d).

In order to extend our investigation of cell reprogramming as part of the DDR response in *Ptch1* ^+/−^ GCPs, we also tested the expression level of the RNA binding protein Lin28 and miR-125b, as it is known that their equilibrium maintains stem cell self-renewal and plasticity or drives neuronal lineage commitment and differentiation^29^. We therefore investigated the expression levels of Lin28 and miR-125b in *Ptch1* ^+/−^ and WT GCPs. As shown in Fig. 3e, Lin28 expression was statistically significantly higher in sham-irradiated *Ptch1* ^+/−^ compared with WT GCPs (*P* < 0.05), and this difference was strikingly increased after exposure to ionizing radiation, especially in combination with *Ptch1* heterozygosity (2.5-fold increase). Accordingly, there was a lower miR-125b expression level (Fig. 3f) in sham-irradiated *Ptch1* ^+/−^ GCPs compared with WT counterparts (*P* < 0.001) and this difference was exacerbated after irradiation (2.3-fold decrease), again indicating a less differentiated phenotype of irradiated *Ptch1* ^+/−^ GCPs.

**Figure 4:**
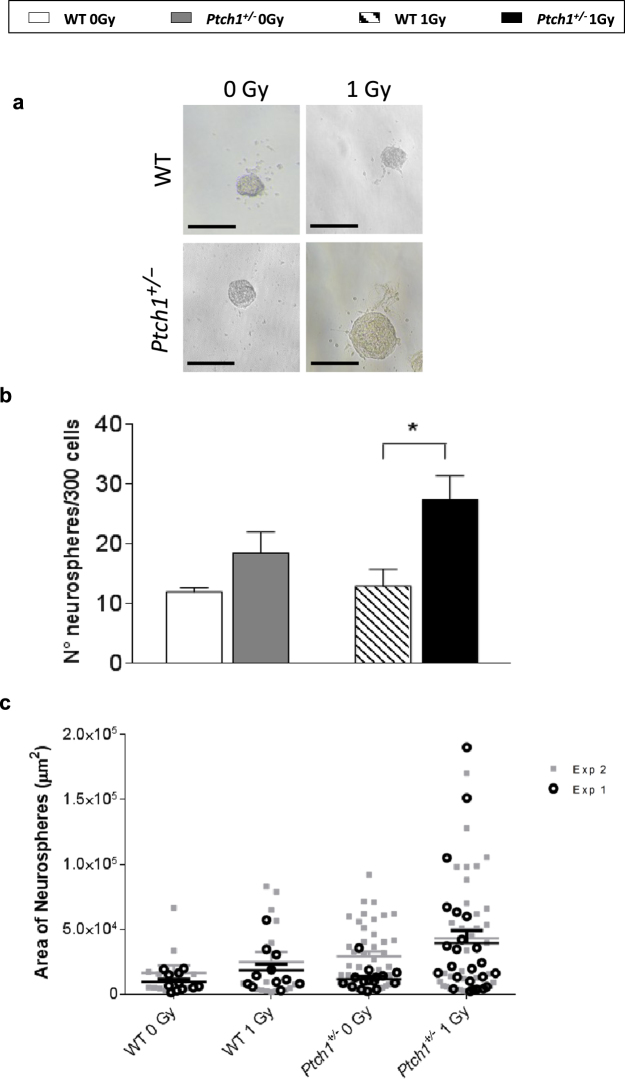
Representative images of neurospheres obtained from 300 seeded WT and *Ptch1* ^+/−^ GCPs in unirradiated and irradiated conditions. Bars = 100 μm. (**a**) The number of neurospheres shown in (**b**) are presented as mean values ± SD of results derived from biological triplicate experiments. Neurosphere areas shown in (**c**) were measured in phase-contrast images using LASCore software and data are presented as mean values ± SD of results derived from two biological replicates. *P ≤ 0.05.

To better characterize dedifferentiated cells, we tested their ability to grow as neurospheres. As shown in Fig. 4b, activation of the Shh pathway was sufficient to increase the number of neurosphere-forming cells compared with WT cells (1.5-fold). Noteworthy, clonogenicity of *Ptch1* ^+/−^ GCPs significantly increased after irradiation (*P* < 0.05). Moreover, after morphometric analysis of neurospheres, we show that sham-irradiated *Ptch1* ^+/−^ GCPs form colonies with larger average area compared to that observed in WT cells (Fig. 4c). Again, this trend was accentuated after irradiation, and we obtained neurospheres with an area >10 μm^2^ only from irradiated *Ptch1* ^+/−^ GCPs.

Altogether, these results support the hypothesis that radiation induces expansion of the stem-like cell compartment through cell reprogramming and self-renewal maintenance, and that this mechanism is strongly enhanced by activation of the Shh signaling pathway.

### Nanog controls proliferation, apoptosis and stemness in irradiated *Ptch1*^+/−^ GCPs

**Figure 5:**
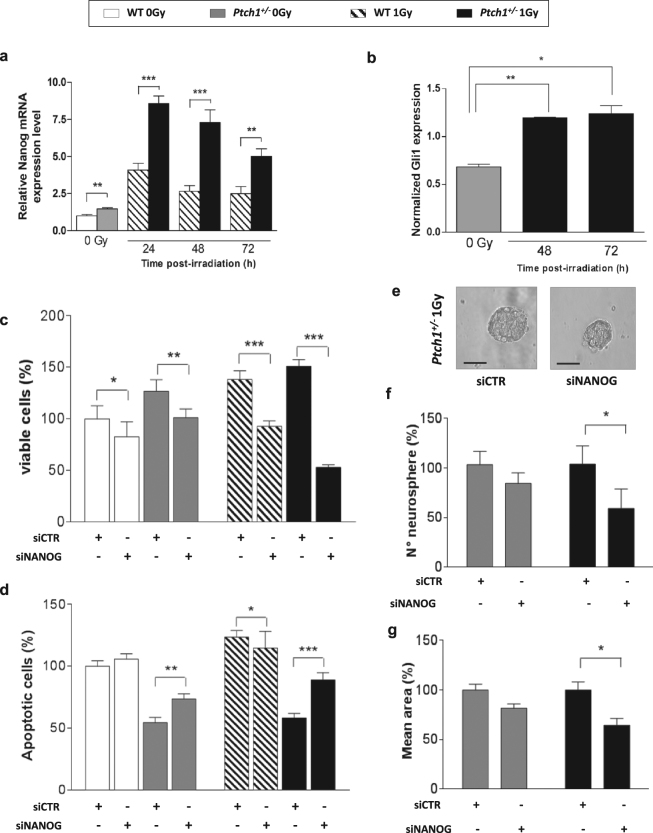
Kinetics of Nanog mRNA expression at different times after irradiation in WT and *Ptch1* ^+/−^ GCPs (**a**). Densitometric analysis of Gli1 protein expression in *Ptch1* ^+/−^ GCPs 48 and 72 hrs post-irradiation (**b**). Cell viability (**c**) and apoptotic assay (**d**) in WT and *Ptch1* ^+/−^ GCPs after transfection with control siRNA (siCTR) or Nanog siRNA (siNanog) in unirradiated and irradiated conditions. Expression levels in WT siCTR untreated cells are taken as 100. (**e–g**) Quantitative and dimensional analysis of neurospheres obtained from unirradiated and irradiated *Ptch1* ^+/−^ GCPs after silencing of Nanog expression (**e**; right panel) or treated with control siRNA (**e**; left panel). Bars = 100 μm. The number of neurospheres (**f**) and their size (**g**) decreased in siNanog *Ptch1* ^+/−^ GCPs compared with siCTR *Ptch1* ^+/−^. Values in siCTR untreated cells are taken as 100. *P ≤ 0.05; **P ≤ 0.01; ***P ≤ 0.001.

Besides the well-recognized role of Nanog as transcription factor in embryonic SCs and in maintaining pluripotency^27,30,31^, Nanog is also a critical mediator of Shh-driven self-renewal of neural SCs. Both Nanog and Gli1 are highly expressed in postnatal cerebellar NSCs and in Shh-dependent mouse and human MB SCs^32^. Therefore, we followed Nanog mRNA expression in GCPs until 72 h, and found it upregulated at 24, 48 and 72 h post-irradiation (Fig. 5a). Similarly, Gli1 expression was upregulated at 48 and 72 h (Fig. 5b). We thus focused on Nanog in our efforts to characterize the dedifferentiation process.

We silenced Nanog expression in our cell lineages (siNanog GCPs). In sham-irradiated condition, we observed statistically significant reduction of cell viability in cultures of both siNanog-WT and -*Ptch1* ^+/−^ GCPs compared with untreated cells (siCTR GCPs). Irrespective of genotype, cell viability strongly decreased after irradiation, although this was much more evident in *Ptch1* mutants (Fig. 5c). Decreased cell viability is attributable to concomitant increased level of apoptosis, as shown in Fig. 5d.

Importantly, the number of neurosphere-forming cells decreased in siNanog *Ptch1* ^+/−^ GCPs compared with siCTR *Ptch1* ^+/−^ in both experimental groups, reaching statistical significance in irradiated condition (Fig. 5f; *P* < 0.05). This effect is also reflected in the lower mean size of neurospheres, as shown in Fig. 5e,g.

### Consequences of radiation-induced expansion of the stem-like cell compartment on cerebellar development and MB onset

**Figure 6:**
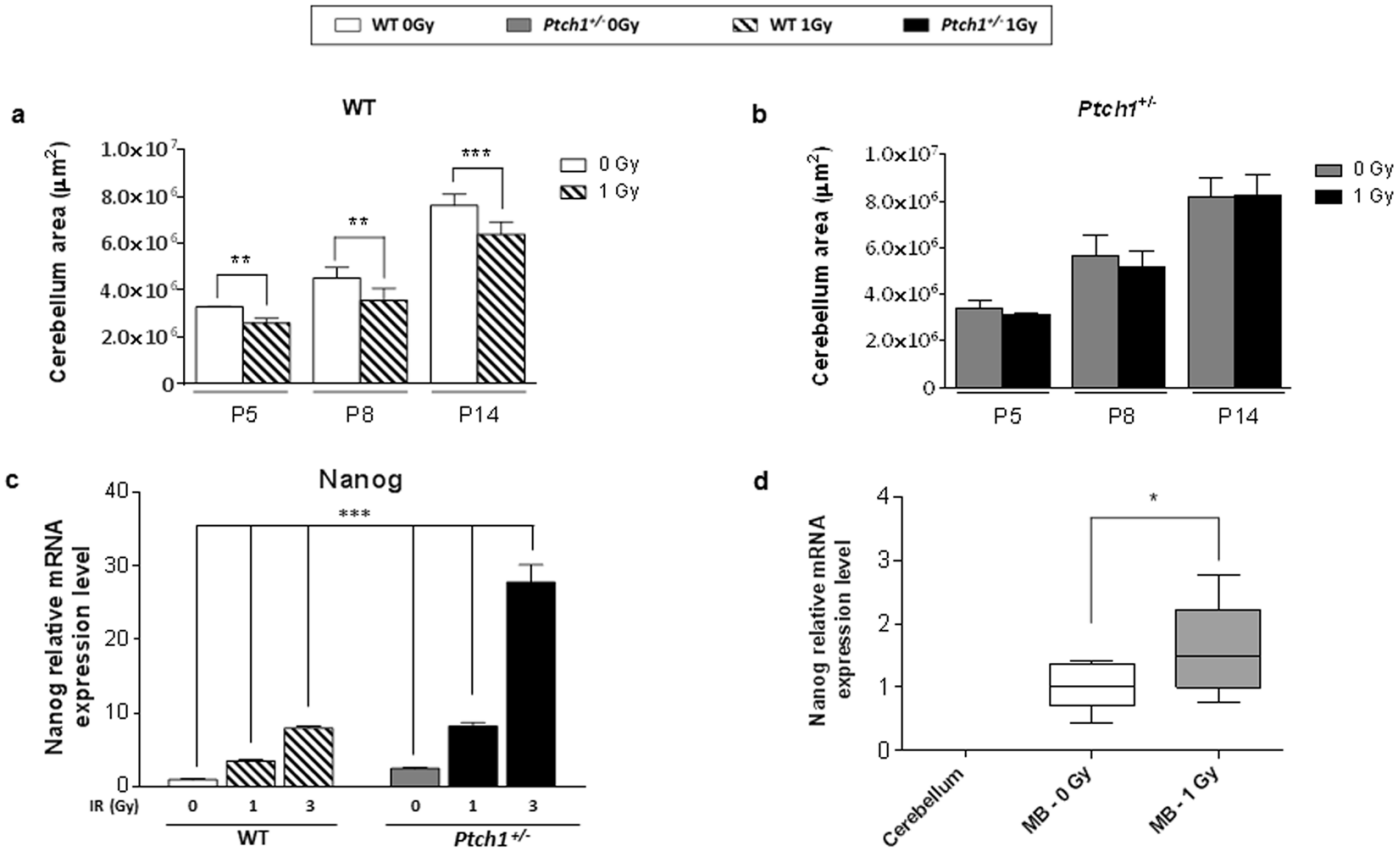
Dimensional analysis of cerebellar midsagittal sections from unirradiated and irradiated WT (**a**) and *Ptch1* ^+/−^ (**b**) mouse cerebella at different ages of postnatal development. (**c**) Relative Nanog mRNA expression levels in WT and *Ptch1* ^+/−^ GCPs at 4 hours after irradiation with increasing doses of X-rays, relative to unirradiated GCPs. (**d**) Relative Nanog mRNA expression levels in the cerebellum of adult *Ptch1* ^+/−^ (n = 4), and in spontaneous (n = 9) and radio-induced (n = 11) MBs. *P ≤ 0.05; **P ≤ 0.01; ***P ≤ 0.001.

To assess whether the different DDR observed *ex vivo* between WT and *Ptch1* ^+/−^ GCPs leads to cerebellar development anomalies *in vivo*, we measured cerebellar area at different ages (P5, P8 and P14) of postnatal development, occurring during the first 21 postnatal days in the mouse^33^. In absence of irradiation, histological examination of cerebellar midsagittal sections from WT and *Ptch1* ^+/−^ mice showed comparable foliation patterns (data not shown); however, as shown in Fig. 6a,b, *Ptch1* ^+/−^ mouse cerebella were larger than WT cerebella at P8 (5.6 *vs* 4.5 × 10^6^; *P* < 0.05) and P14 (8.2 *vs* 7.6 × 10^6^; *P* < 0.05). These data, in agreement with the higher proliferation index of *Ptch1* ^+/−^ GCPs (see Supplementary Information file (Table S1)), underline that Shh activation, while not altering the basic foliation pattern, increases the overall size of the cerebellum, as already reported by other authors^34^.

After irradiation, WT mice showed a statistically significant decrease of cerebellum area compared with age-matched sham-irradiated mice at all time-points (Fig. 6a). Of note, no statistically significant dimensional difference was found in *Ptch1* ^+/−^ mouse cerebella after IR-exposure (Fig. 6b). These results strongly suggest that the expansion of the stem-like compartment and the concomitant inhibition of radiation-induced apoptosis observed in *Ptch1* ^+/−^ GCPs after irradiation counteracts the cerebellar reduction by radiation, as also observed in rats^35^.

On the other hand, the Nanog-driven expansion of a stem-like cell compartment which displays genomic instability due to the accumulation of DNA damage (see Fig. 1A), could be strictly related to the dramatically high MB incidence typical of the *Ptch1* ^+/−^ mouse model in response to radiation^18,19^.

As Nanog mediates GCPs response to radiation, we asked whether its expression could be dose dependent. Therefore, we also measured Nanog expression in WT and *Ptch1* ^+/−^ GCPs irradiated with 3 Gy. As shown in Fig. 6c, a statistically significant dose-dependent increase of Nanog mRNA was found in WT cells. In *Ptch1* ^+/−^ GCPs this dose dependence was more marked compared with WT GCPs. We then checked Nanog expression in spontaneous (n = 9) and radio-induced (n = 11) MBs, as well as in adult cerebellum (n = 4) for comparison. As shown in Fig. 6d, the mean value of Nanog expression in radio-induced MBs was statistically significantly higher compared with that found in spontaneous tumors (*P* < 0.05). As expected, no Nanog expression was detected in adult cerebellum.

## Discussion

Tumorigenesis is thought to depend on capacity for long-term self-renewal. During mouse normal cerebellum development, GCPs do not exhibit this capacity: they proliferate for 2–3 weeks after birth, then exit the cell cycle and undergo terminal differentiation. Deletion of one copy of *Ptch1* increases proliferation of GCPs^12–14^ but many of these are still able to differentiate and only a subset of them continues to proliferate and form tumors with a spontaneous MB rate of 8% on CD1 genetic background^18^. One explanation for this is that full transformation of GCPs requires changes that only occur in a subset of GCPs.

The aim of our study was the clarification of mechanisms that underlie Shh-dependent radio-induced MB tumorigenesis, and for this we took advantage of the use of highly radiosensitive *Ptch1* ^+/−^ mice^16,17^. In particular, the present study addresses the question of the effects of DDR on GCPs self-renewal, with particular emphasis on the role of Nanog in GCPs dedifferentiation and DDR processing by highly purified GCPs irradiated *ex vivo*.

To explore how deregulated Shh signaling and tissue injury caused by radiation contribute to MB tumorigenesis, we first investigated the accumulation of γ-H2AX, a biomarker of DNA double strand breaks, in WT and *Ptch1* ^+/−^ GCPs at different time points after irradiation and in respective control cells. We detected higher levels of γ-H2AX-positive cells in *Ptch1* ^+/−^ compared with WT GCPs, confirming that Shh signaling deregulation is able to drive towards an unstable cell phenotype favoring accumulation of genomic alterations^24^. Despite higher levels of DNA damage, we found that *Ptch1* ^+/−^ GCPs were less prone to spontaneous and radio-induced apoptosis, showed higher expression of the survival factor Bcl2, and higher proliferation rates than WT GCPs. This might be due to impairment in radiation–induced checkpoint activation^24^. The cellular response to DNA damage usually depends on the tumor suppressor p53^36^. In agreement with our previous *in vivo* data^19^, we report here that *ex vivo* irradiation of *Ptch1* ^+/−^ GCPs does not activate p53. The lack of p53 activation after ionizing radiation, combined with weak activation of p21, may suggest dedifferentiation of GCPs to a stem-like cell phenotype leading to cell-cycle entry and self-renewing divisions, as it usually happens in SCs^26^.

In agreement, increased levels of Lin28 associated with decreased expression of miR-125b after radiation treatment, strongly suggest a less differentiated status of *Ptch1* ^+/−^ compared with WT GCPs. Notably, the expression of factors that are known to induce stem-like properties in differentiated cells, i.e., OCT4, Nanog and Sal4^28,37,38^, were also significantly more expressed in *Ptch1* ^+/−^ GCPs with respect to control cells. Also, their expression was higher compared with irradiated WT GCPs, showing that Shh deregulation and irradiation synergize to amplify the stem cell compartment. In support of these molecular results, clonogenicity of *Ptch1* ^+/−^ GCPs significantly increased after irradiation.

Our data suggest that endogenous activation of both Oct4 and Nanog expression could have a role in the reprogramming process induced by irradiation. Of note, Nanog is a critical mediator of Shh-driven self-renewal of NSCs and its transcriptional activation is directly sustained by Gli1 and Gli2^32^; moreover, Zbinden and colleagues^39^ demonstrated that Nanog is situated in a neat balance between p53 and Shh signaling, whose deregulation results in an aberrant expression of Nanog. Focusing on Nanog we show, for the first time, that Nanog depletion abrogates the enhancing response to radiation controlling cell viability, apoptosis, cell reprogramming and cell-renewal of *Ptch1* ^+/−^ GCPs.

Although in *Ptch1* mutant cells all biological endpoints analyzed are magnified by an aberrant Shh pathway activation, irradiation similarly affects WT GCPs, highlighting a general cell response to the radiogenic insult. However, at this stage of postnatal development GCPs are, independently by genotype, strongly influenced by Shh pathway; moreover, in our *ex-vivo* experimental set-up, GCPs viability is maintained only adding Shh to the medium. Consequently, our results highlight a synergic interaction between Shh pathway and radiation in driving cell reprogramming and self-renewal maintenance, through Nanog activation.

Self-renewal in neural progenitor cells has been previously described in response to appropriate signals and factors such as proto-oncogenes Myc and Bm1^40,41^ and the Wnt signaling^42^. These results, focused on the knowledge of molecular mechanisms controlling the induction of neural progenitor cells, provide a near limitless source of neural cells for cell-replacement therapies *in vivo* and cell-based *in vitro* models of neurological disease. On the contrary, little information is available on somatic normal cell dedifferentiation after exposure to physical stress.

Many studies have underlined that radiation-induced injury in stem cells may closely associate with future cancer risks^43–45^. Thus, the Nanog-driven expansion of a highly unstable stem-like compartment, due to the accumulation of DNA damage, could be strictly related to the dramatically high MB incidence characteristic of the *Ptch1* ^+/−^ mouse model in response to radiation^18^. In support of this, we report a higher Nanog expression in radio-induced MBs compared with spontaneous ones. Moreover, a key role of the miR-17–92 cluster family in the control of MB progression has been shown^46^. Significant differences in miR-19a expression emerged between radio-induced and spontaneous MB in our previous work^23^. These data strengthen our conclusions on the role of Nanog in driving radiation-induced tumorigenesis, being the cluster miR-17-92 a direct transcriptional target of Nanog^47^. Importantly, the dose-dependent increase of Nanog expression in GCPs closely reflects the well-described dependence on dose of MB incidence in *Ptch1* ^+/−^ mice^48^.

**Figure 7:**
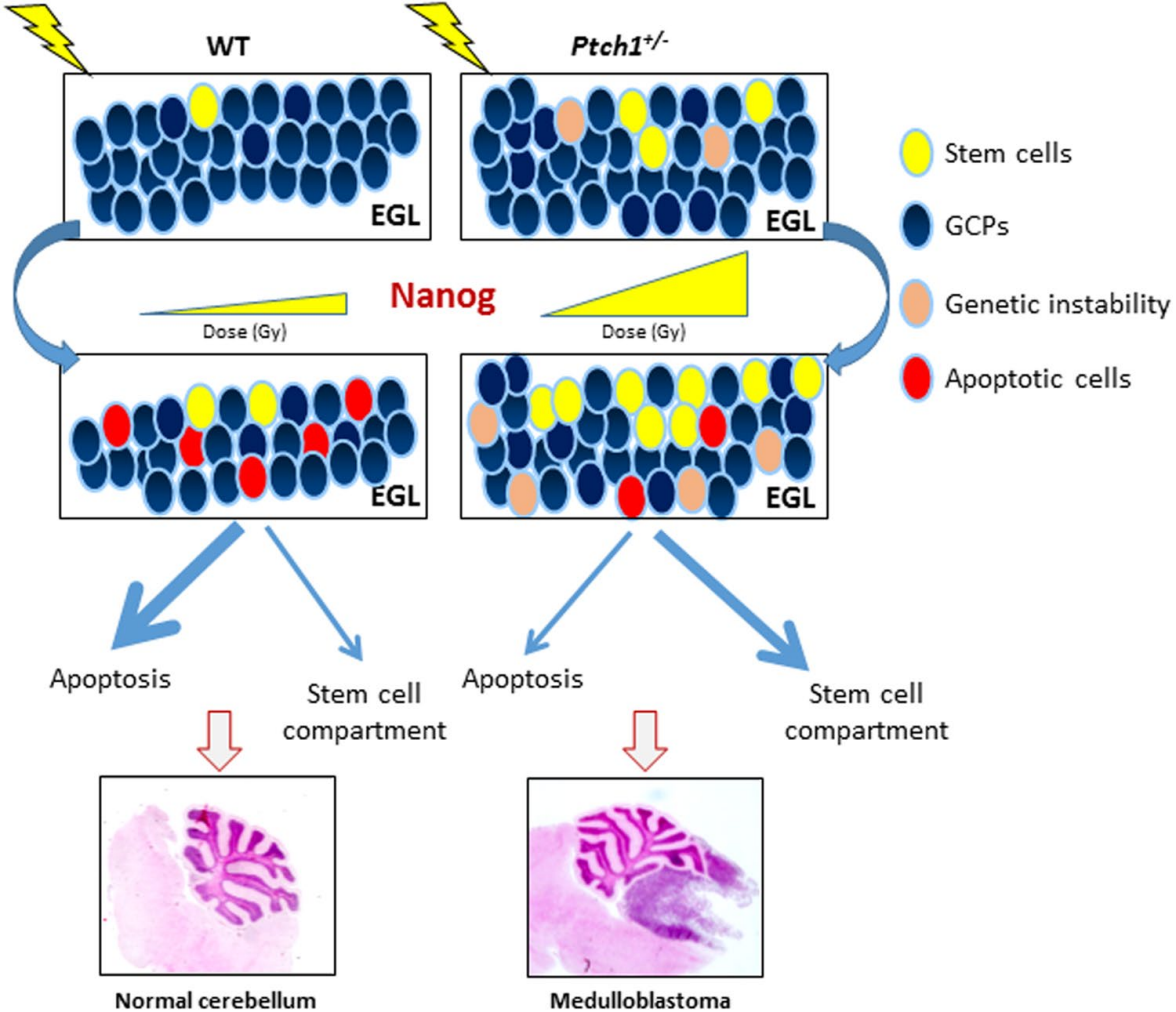
Graphic highlight of results. Nanog controls the DNA damage response of GCPs, influencing also the expansion of the stem-like cell compartment through cell-reprogramming and self-renewal maintenance after radiogenic insult. In *Ptch1-*mutated cells, the magnified Nanog-driven expansion of a highly unstable stem-like cell compartment, due to the accumulation of DNA damage, could be strictly related to the dramatically high MB incidence that characterizes the *Ptch1* ^+/−^ mouse model in response to radiation.

In conclusion, our study provides original insights, summarized in Fig. 7, in the interaction between radiation exposure and Shh-deregulation in sustaining a phenotype shift toward stemness in the Shh-MB cell-of-origin. Importantly, we show, for the first time, that Nanog depletion abrogates the response to radiation, controlling cell viability and apoptosis as well as clonogenicity of *Ptch1* ^+/−^ GCPs. These results help clarify the complex molecular mechanisms that control radiation-induced Shh-mediated tumorigenesis. Moreover, as aberrant activation of SHH pathway is responsible for ~25–30% of sporadic human MB^49^, for which radiotherapy is an essential component of multimodal treatment, our results might also aid development of new therapeutic strategies to overcome SHH-MB radioresistance, with Nanog as a potential target.

## Methods

### GCPs isolation and irradiation

GCPs were purified from WT and *Ptch1* ^+/−^ mouse cerebella at P2 and maintained in culture as described^23^. Cells were irradiated with 1 Gy of X-rays or sham-irradiated using a Gilardoni CHF 320 G X-ray generator (Gilardoni S.p.A., Mandello del Lario, Italy) operated at 250 kVp, with HVL = 1.6 mm Cu (additional filtration of 2.0 mm Al and 0.5 mm Cu).

Animal studies were performed according to the European Community Council Directive 2010/63/EU and were approved by the local Ethical Committee for Animal Experiments of the ENEA.

### Neurosphere assay

Cells were cultured as neurospheres in selective medium DMEM/F12 supplemented with 0.6% glucose, 25 mg/ml insulin, 60 mg/ml N-acetyl-L-cysteine, 2 mg/ml, heparin, 20 ng/ml EGF, 20 ng/ml bFGF (Peprotech, Rocky Hill, NJ), penicillin-streptomycin and B27 supplement without vitamin A. For the neurosphere-forming assay, cells were plated at clonal density (1–2 cells/mm^2^) into 96-well plates and cultured in selective medium as described above. Unless otherwise indicated, media and supplements for cell culture were purchased from Gibco-Invitrogen (Carlsbad, CA) and chemicals were purchased from Sigma-Aldrich (St Louis, MO).

For the morphometric analysis of neurospheres, images were captured with a Leica digital camera, and analyzed by image analysis software LASCore (Leica Microsystems, Milan, Italy).

### Flow Cytometric Analysis

Briefly, two million cells were fixed with 1 ml Fixation/Permeabilization solution (BD Biosciences, San Jose, CA), vortexed and incubated at 4 °C. Cells were centrifuged, and pellets were resuspended in 1 ml of PI/RNase staining buffer (BD Biosciences). Samples were analyzed by flow cytometry using a FACS Calibur flow cytometer (BD Biosciences). For each sample, at least 1 × 10^5^ cells were analyzed. Cell cycle distribution and hypodiploid DNA content were calculated by Cell Quest software (BD Biosciences). For endogenous visualization of γ-H2AX by FACS, we used anti-phospho γ-H2AX, (Ser139) (# 16–202, Upstate Biotechnology Inc., Lake Placid, NY).

### RNA isolation and real-time qPCR

RNA isolation from cells and MBs (obtained from the ENEA archive of frozen tumors) was performed with miRNeasy Mini Kit (# 217004; QIAGEN, Milan, Italy.) After quantification, 2 μg of total RNA was reverse transcribed with High-Capacity cDNA Reverse Transcription Kit (Applied Biosystems, Foster City, CA), and qPCR was carried out by StepOnePlus™ Real-Time PCR System (Applied Biosystems) using Power SYBR® Green PCR Master Mix (Applied Biosystems). Oligonucleotide primers used for quantitative RT-PCR are listed in Supplementary Information file (Table S2). Reactions were performed in triplicate from each biological replicate. Relative gene expression was quantified using Glyceraldehyde-3-phosphate dehydrogenase (Gapdh) as house-keeping gene. The ΔΔCt quantitative method was used to normalize expression of the reference gene and to calculate the relative expression levels of target genes.

miRNA expression was performed as described earlier^23^; analysis was carried out with TaqMan® miRNA Assay (ThermoFisher Scientific, Milan, Italy) for hsa-miR-125b-5p (# A25576 Assay ID: 477885_mir) and for U6 snRNA (# Assay ID: 001973).

### Western blot assay

Cells were lysed with T-PER® Tissue Protein Extraction Reagent (# 78510; Pierce Biotechnology, Rockford, IL) added with protease inhibitors. Lysates were separated on pre-cast gels (Bio-Rad Laboratories; Hercules, CA) and immunoblotted using standard procedures. Anti-p21 (C19) (# sc-397; Santa Cruz Biotechnology, Santa Cruz, CA), anti-Bax (N20) (# sc493, Santa Cruz Biotechnology), anti Bcl-2 (N19) (# sc-492, Santa Cruz Biotechnology), anti-Gli1 (# NB600-600, Novus Biologicals, Littleton, CO, USA) and HRP-conjugated secondary antisera (Santa Cruz Biotechnology) were used followed by enhanced chemiluminescence (ECL Amersham, Amersham, UK). Densitometry calculations for western blot were calculated using ImageJ software, verifying for non-saturation and subtracting background. Band intensities were sampled three times and normalized against HSP90 (# 4874; Cell Signaling Technology Inc., Danvers, MA).

### Silencing of Nanog in GCPs

Transfection of siRNA duplexes (40 nM) directed against the Nanog mRNA coding sequence (siGENOME Mouse Nanog (71950) siRNA SMARTpool; Dharmacon, Thermo Fisher Scientific, Waltham, MA) was carried out using the INTERFERin™ siRNA Transfection Reagent (Polyplus Transfection, New York, NY) according to the manufacturer’s instructions. Control transfections were carried out with a pool of validated siRNA controls (siGENOME Non-Targeting siRNA Pool #1, D-001206-13-20, Dharmacon). GCPs were isolated at P2 and transfected with siRNAs the same day, the following day cells were seeded and irradiated.

### Viability and apoptosis

Viability and apoptosis of GCPs were performed using CellTiter-Glo® Luminescent Cell Viability Assay (# G7570; Promega Madison, WI, USA) and Caspase-Glo® 3/7 Assay (# G8091; Promega) according to the manufacturer’s instructions.

### Morphometric Analysis

Brains (n = 6 for each genotype) were collected from unirradiated and 1 Gy- irradiated WT and *Ptch1* ^+/−^ mice at different experimental time points (i.e., postnatal day (P) 5, P8 and P14), fixed in 10% buffered formalin, embedded in paraffin wax according to standard techniques, sectioned and stained with hematoxylin/eosin for histology. Morphometric analysis to measure cross sectional area was carried out using imaging software NIS-Elements BR 4.00.05 (Nikon Instruments Europe B.V., Italy).

### Statistical analysis

All quantitative data are presented as mean ± SD. When both genotype and dose effects were analyzed, the level of significance was determined by two-way analysis of variance with Bonferroni post-hoc tests to compare replicate means (GraphPad Software, San Diego, CA, USA). If not otherwise stated, statistical significance (*P*) was calculated by two-tailed Student’s *t*-test.

## Electronic supplementary material

Supplementary files
