# Supplementary material for: Nanog-driven cell-reprogramming and self-renewal maintenance in Ptch1+/− granule cell precursors after radiation injury
Source: Sci Rep. 2017 Oct 27;7:14238. doi: 10.1038/s41598-017-14506-6 (PMC5660207; doi:10.1038/s41598-017-14506-6)
Supplement: Supplementary file 1 — Supplementary files [file 41598_2017_14506_MOESM1_ESM.pdf]

Nanog-driven cell-reprogramming and self-renewal maintenance in *Ptch1*$^{+/-}$ granule cell precursors after radiation injury

Barbara Tanno[1], Simona Leonardi[1], Gabriele Babini[2], Paola Giardullo[3,4], Ilaria De Stefano[3], Emanuela Pasquali[1], Anna Saran[1*] and Mariateresa Mancuso[1*]

[1]Laboratory of Biomedical Technologies, Italian National Agency for New Technologies, Energy and Sustainable Economic Development (ENEA), Rome, Italy; [2]Department of Physics, University of Pavia, Pavia, Italy; [3]Department of Radiation Physics, Guglielmo Marconi University, Rome, Italy; [4]Department of Sciences, Roma Tre University, Rome, Italy;

Supplementary figures, tables and figure legends

Table S1. Cell cycle analysis of unirradiated and irradiated WT and *Ptch1*$^{+/-}$ GCPs.

Mice	Irradiation (Gy)	Time (h)	G0 (%)	G1 (%)	Proliferation index (%)
	-	0	2.74	76.61	20.65
	1	4	14.82	62.23	22.95
	1	24	17.57	70.98	11.46
	-	0	1.62*	74.37*	24.10*
	1	4	11.87*	62.03	25.70*
	1	24	9.23*	75.32*	14.94*

DNA content of GCPs was measured by propidium iodide staining followed by flow cytometric analysis. Each phase of the cell cycle was expressed as percentage of the complete cell cycle. Values are representative of three independent experiments. *$P < 0.05$ *Ptch1*$^{+/-}$ *vs* WT GCPs at the same time-point.

Supplementary Table S1

Figure S1: Uncropped Western blots related to main Fig. 2.

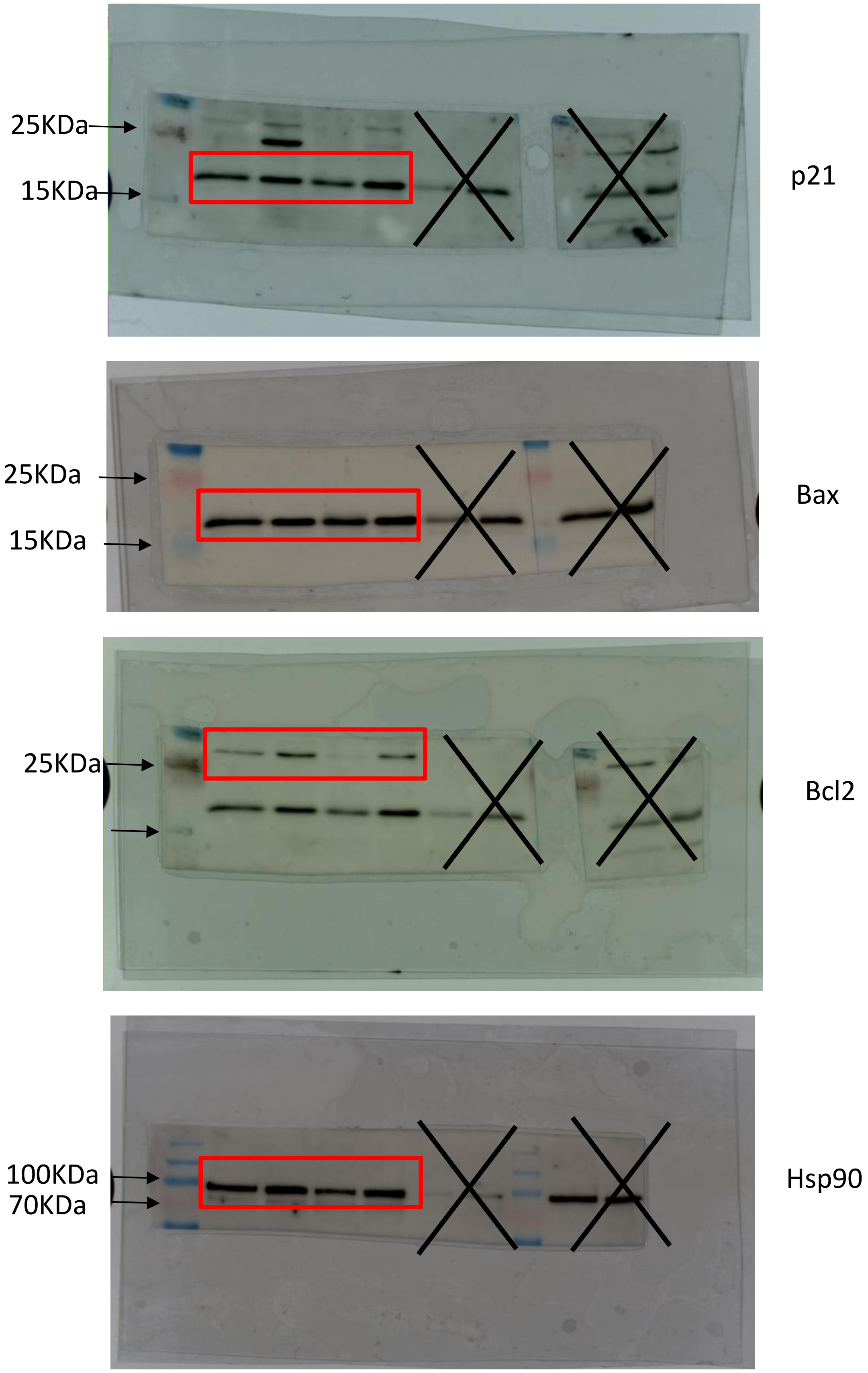

Supplementary Figure S1

Table S2. Primer sequences used for qPCR

Gene	Forward primer	Reverse primer
Nanog	5'-GCTCAGCACCAGTGGAGTATCC-3'	5'-TCCAGATGCGTTCACCAGATAG-3'
Sox2	5'-CGCGGCCGGTATTTATAATC-3'	5'-ACTTTTGTCCGAGACCGAGAA-3'
Oct4	5'-AAAGCCCTGCAGAAGGAGCTAGAA-3	5'-AACACCTTTCCAAAGAGAACGCCC-3'
Sal4	5'CTCCAGCAAATCCAGCTTACG3'	5'GCGCTGCCCACATGTTC3'
p53	5' TGCATGGACGATCTGTTGCT3'	5' TTCACTTGGGCCTTCAAAAAA3'
p21	5'CGAGAACGGTGGAACTTTGAC3'	5'CAGGGCTCAGGTAGACCTTG3'
Lin28	5' AGTCTGCCAAGGGTCTGGAA-3'	CGCTCACTCCCAATACAGAACA-3'
Gapdh	5'-CATGGCCTTCCGTGTTCCTA-3'	5'-GCGGCACGTCAGATCCA-3'

Supplementary Table S2

Figure S2: Uncropped Western blots related to densitometric analysis of Gli1 showed in Fig. 5b.

250Kda
130Kda
95Kda
72Kda

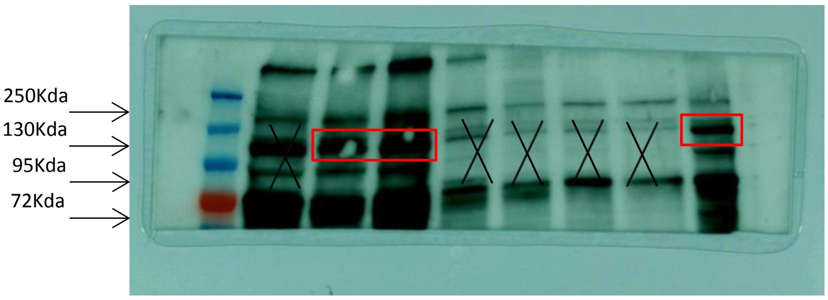

Gli1

250Kda
130Kda
95Kda
72Kda

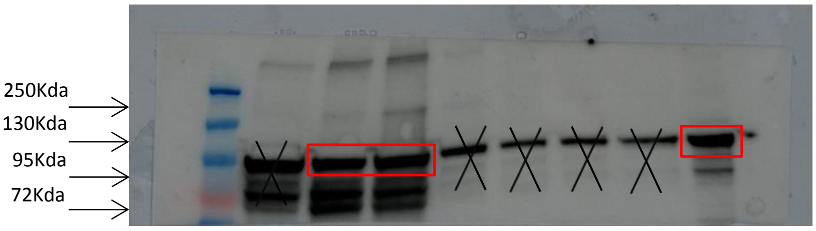

Hsp90

Supplementary Figure S2
